# Idiopathic isolated adrenocorticotropic hormone deficiency combined with testicular germ cell tumor: Case report

**DOI:** 10.1097/MD.0000000000045585

**Published:** 2025-10-31

**Authors:** Ming Yang, Shuangzhu Lin, Dongting Fu, Yu Wang, Siyu Lu, Man Li, Xiaoli Wang

**Affiliations:** a Department of Endocrinology and Metabolism, The Affiliated Hospital to Changchun University of Chinese Medicine, Changchun, China; b Changchun University of Chinese Medicine, Changchun, China; c Department of Endocrinology and Metabolism, Institute of Endocrinology, NHC Key Laboratory of Diagnosis and Treatment of Thyroid Diseases, The First Affiliated Hospital of China Medical University, Shenyang, China.

**Keywords:** adrenocorticotropic hormone, alopecia areata, gonadotropin-releasing hormone, paraneoplastic syndromes, testicular neoplasms

## Abstract

**Rationale:**

Idiopathic isolated adrenocorticotropic hormone (ACTH) deficiency (IAD) is a rare disorder. Its clinical presentation is nonspecific, with primary manifestations including fatigue, weakness, nausea, vomiting, hyponatremia, hypoglycemia, and neuropsychiatric symptoms. This case report describes a patient with IAD who presented with alopecia areata as the initial manifestation, concurrent with a testicular germ cell tumor. To the best of our knowledge, this represents the first reported case of IAD presenting initially with alopecia areata, and an association with testicular germ cell tumors has not been previously described.

**Patient concerns:**

A 50-year-old male presented with a significant reduction in scalp hair density, a decrease in the rest of the body’s hair, whitening of new hair growth, loss of libido, fatigue, and poor mental health.

**Diagnoses:**

The patient was diagnosed with idiopathic isolated adrenocorticotropic hormone (ACTH) deficiency (IAD) and testicular germ cell tumor.

**Interventions:**

The patient was treated with oral glucocorticoids and the testicular germ cell tumor was successfully resected.

**Outcomes:**

At follow-up after 2 months, the patient’s clinical symptoms improved significantly, ACTH and cortisol levels returned to near normal on recheck, estrogen levels decreased to normal, and glucocorticoids were discontinued with no significant adverse events.

**Lessons:**

This report suggests that there may be a potential association between alopecia areata, IAD, and testicular germ cell tumors and that the pathogenesis may be related to the autoimmune response induced by paraneoplastic syndrome. Clinically, patients with IAD of unknown etiology need to be carefully screened for the possibility of neoplastic disease in all systems.

## 1. Introduction

Idiopathic isolated adrenocorticotropic hormone deficiency (IAD) is a rare disorder characterized by the selective involve of the hypothalamic-pituitary-adrenal (HPA) axis, manifesting as reduced serum cortisol levels and absolute or relative adrenocorticotropic hormone (ACTH) deficiency, with no involvement of other pituitary axes. Clinically, IAD is divided into 2 subtypes: congenital and acquired. The congenital type is mostly seen in children and is related to gene variants, while the pathogenesis of the acquired type is still unclear, and most scholars consider it to be closely related to autoimmunity.^[1,2]^ IAD typically presents with nonspecific clinical manifestations, including fatigue, weakness, nausea, vomiting, hyponatremia, hypoglycemia, and neuropsychiatric symptoms. Owing to this atypical presentation, IAD is frequently misdiagnosed as a gastrointestinal, neurological, or psychiatric disorder.^[3]^

Herein, we report a case of adult-onset idiopathic isolated ACTH deficiency, in which alopecia areata served as the initial symptom, accompanied by a testicular germ cell tumor. A gonadotropin-releasing hormone (GnRH) excitability test and immunohistochemistry effectively helped in the diagnosis of the disease during the management of this patient.

To the best of our knowledge, no previous reports have described IAD with alopecia areata as the presenting symptom. Additionally, IAD as a paraneoplastic syndrome is rarely documented in the literature. Notably, the limited reported cases of paraneoplastic IAD are primarily associated with malignancies such as small-cell lung cancer, thymic carcinoma, and neuroendocrine carcinoma. An association between IAD and testicular germ cell tumors has not been reported in the existing medical literature. Given the rarity and unique clinical features of this case, further research is warranted to elucidate the potential relationships among alopecia areata, IAD, and testicular germ cell tumors.

## 2. Case report

In June 2024, a 50-year-old male patient was presented to the dermatology department with a sudden reduction in hair volume and whitening of newly grown hair. He was clinically diagnosed with alopecia areata. The patient received treatment with compound betamethasone (intralesional injection) and topical 5% minoxidil tincture. However, after half a year of observation, no significant clinical improvement was achieved. During the treatment course, the patient’s alopecia areata symptoms gradually worsened, and he also developed symptoms of decreased libido, fatigue, and poor mental state. At the initial consultation with the endocrinology department in December 2024, the patient complained of significantly reduced scalp hair density, decreased hair on other parts of the body, whitening of newly grown hair and eyebrows, along with decreased libido, fatigue, and mental lassitude. He additionally reported newly onset palpitations, excessive sweating, memory impairment, and poor sleep quality in recent periods. No symptoms of anorexia, nausea, vomiting, or weight loss were observed.

The patient had a history of good health. He denied a history of hypertension, coronary heart disease, hepatitis, tuberculosis, drug allergies, surgery, trauma, meningitis, or neoplastic diseases. Furthermore, he denied a family history of genetic disorders.

### 2.1. Physical examination

The patient’s vital signs were stable: body temperature 36.2°C, heart rate 78 beats/min, respiratory rate 18 breaths/min, and blood pressure 135/90 mm Hg. He had a medium build with normal physical development, without central obesity, plethoric facies, dysmorphic facial features, or dorsocervical fat pad. Dermatological examination showed alopecia totalis, near-complete eyebrow loss, and newly grown hair/eyebrows that were white, fine-textured, and sparsely distributed. No abnormalities were found in the skin or mucous membranes, and there was no hyperpigmentation or hypopigmentation. No acanthosis nigricans was observed on the posterior neck. Thyroid examination revealed no obvious abnormalities, and there was no generalized fine tremor. Breast examination showed symmetrical development, with no palpable masses or nipple discharge. Abdominal examination detected no purple striae. Both testes were normal in size, with no abnormal findings in the testes or scrotum. No skeletal deformities were noted. Muscle strength and tone were normal in all extremities.

### 2.2. Laboratory findings

#### 2.2.1. Hormonal assays

ACTH levels were markedly reduced across the diurnal cycle: 7.13 pg/mL at 08:00, 1.00 pg/mL at 16:00, and 2.23 pg/mL at 24:00 (reference range: 7.2–63.3 pg/mL). Cortisol (COR) maintained a normal diurnal rhythm but was globally suppressed (Table 1). Additionally, estradiol was elevated at 268.00 pmol/L (reference range: 73.4–206 pmol/L).

**Table 1:** Laboratory data of ACTH and COR levels.

Items	08:00 am	15:00 pm	24:00 pm	Normal range
ACTH (pg/mL)	7.13	1.00	2.23	7.2–63.3
COR (nmol/L)	154.70	49.78	18.21	133–537

ACTH = adrenocorticotropic hormone, COR = cortisol.

Other pituitary-related hormones were within normal ranges, including testosterone (TES), prolactin (PRL), luteinizing hormone (LH), follicle-stimulating hormone (FSH), thyroid-stimulating hormone, free triiodothyronine (FT3), free thyroxine (FT4), antithyroid peroxidase antibody (TPOAb), antithyroglobulin antibody (TGAb), thyroid-stimulating hormone receptor antibody (TRAb), androstenedione (AND), dehydroepiandrosterone (DHS), free testosterone (F-TES), sex hormone-binding globulin, progesterone (PRG), and human chorionic gonadotropin.

#### 2.2.2. Routine and biochemical tests

Fasting blood glucose 4.4 mmol/L (reference range: 3.9–6.1 mmol/L); serum potassium 4.24 mmol/L (3.50–5.30 mmol/L); serum sodium 142.0 mmol/L (137.0–147.0 mmol/L).

Routine blood, urine, and stool tests; liver and renal function; electrolytes; glycated hemoglobin; and tumor markers (carcinoembryonic antigen, carbohydrate antigen 125, carbohydrate antigen 153, carbohydrate antigen 199, and alpha-fetoprotein) were all normal.

### 2.3. Imaging examinations

**Figure 1.**
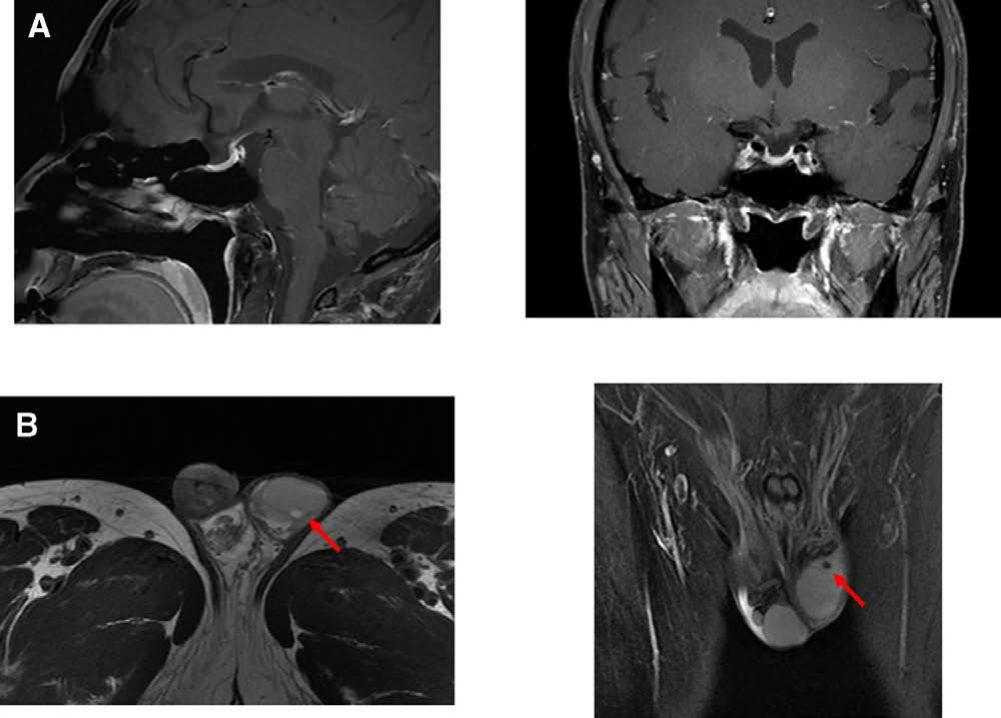
Imaging findings of the pituitary gland and left testis. (A) Contrast-enhanced pituitary MRI demonstrating an empty sella turcica. (B) Contrast-enhanced testicular MRI identifying a space-occupying lesion in the left testis (red arrow). MRI = magnetic resonance imaging.

Contrast-enhanced pituitary magnetic resonance imaging (MRI) demonstrated an empty sella turcica (Fig. 1A). Contrast-enhanced computed tomography of the adrenal glands showed no significant abnormalities. Electrocardiography (ECG) and chest computed tomography scans were unremarkable. Ultrasonography of the thyroid gland, liver, gallbladder, spleen, kidneys, and urinary bladder revealed no abnormal findings. Testicular ultrasound identified a hyperechoic focus (approximately 0.89 × 0.55 cm) in the left testis. Contrast-enhanced testicular MRI further revealed a round nodule (≈0.5 cm in diameter) in the left testis with long T1 and mixed T2 signals, and no definite enhancement after contrast administration (Fig. 1B), suggesting a testicular space-occupying lesion.

### 2.4. Diagnosis

The patient presented with markedly reduced ACTH levels, globally low cortisol levels, and normal concentrations of other pituitary-related hormones – findings suggestive of a HPA axis disorder. To clarify the diagnosis, a gonadotropin-releasing hormone (GnRH) stimulation test was performed (Table 2). Following intravenous administration of 100 µg GnRH, LH and FSH responses were monitored, confirming normal function of the hypothalamic-pituitary-gonadal axis. Based on the above findings, the patient was diagnosed with IAD.

**Table 2:** Gonadotropin-releasing hormone stimulation test.

	0 min	15 min	30 min	60 min	90 min	120 min
LH (mIU/L)	1.30	18.50	23.70	17.70	15.00	12.00
FSH (mIU/L)	4.93	7.74	9.23	8.71	8.77	8.39

FSH = follicle-stimulating hormone, LH = luteinizing hormone.

### 2.5. Treatment and follow-up

The patient was initiated on oral hydrocortisone replacement therapy, with a regimen of 20 mg at 07:00 and 10 mg at 15:00 daily. Dosage doubling was recommended during periods of stress.

**Figure 2.**
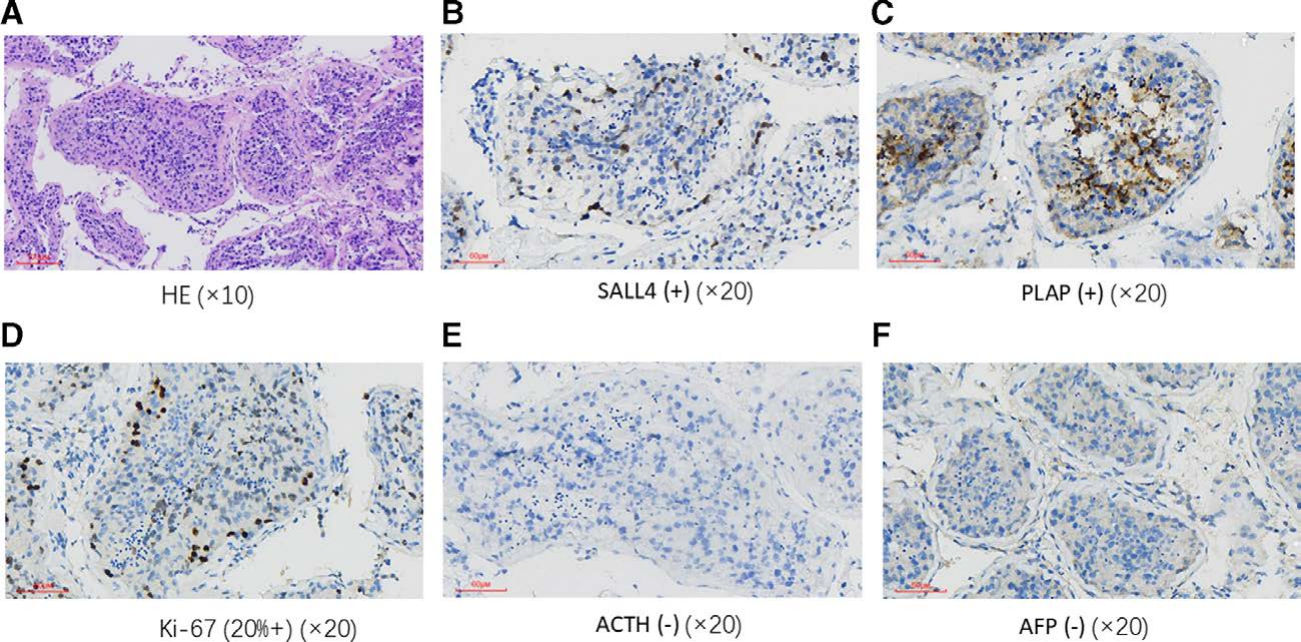
Histopathological and immunohistochemical staining of the left testicular tumor (representative fields). (A) HE staining (×10); (B) SALL4 positive (+) (×20); (C) PLAP positive (+) (×20); (D) Ki-67 proliferation index showing significant elevation (20%+) (×20); (E) ACTH negative (−) (×20); (F) AFP negative (−) (×20). ACTH = adrenocorticotropic hormone, AFP = alpha-fetal protein, HE = hematoxylin-eosin, PLAP = placental alkaline phosphatase, SALL4 = spalt like transcription factor 4.

In January 2025, the patient underwent surgical resection of the left testicular germ cell tumor. Immunohistochemical analysis showed positive staining for Ki-67 (20%+), SALL4 (+), and PLAP (+), with negative staining for ACTH (−) (Fig. 2).

At the first outpatient follow-up in February 2025, the patient reported discontinuing hormone therapy prior to blood sampling. Laboratory tests showed normalized hormone levels: ACTH increased to 17.10 pg/mL, cortisol (COR) recovered to 219.50 nmol/L (133–537 nmol/L), and estradiol normalized at 190.00 pmol/L (73.4–206 pmol/L). Clinically, no further reduction in scalp or body hair was observed, and a small number of new black hairs had emerged. The patient also reported restored libido, normalized physical strength, improved mental state, resolution of palpitations and excessive sweating, and better sleep quality. Subsequently, the patient was advised to attend regular outpatient follow-up visits every 3 months.

## 3. Discussion

IAD is a rare pituitary endocrine disorder in adults, first described by the Japanese researcher in 1954.^[4]^ To date, Japan has reported the highest number of cases globally, with over 300 documented instances. However, comprehensive epidemiological data on IAD remain lacking worldwide, as the existing literature is predominantly limited to case reports and small-scale case series. The precise etiology of IAD remains elusive, though autoimmune mechanisms are widely hypothesized to play a central role. Emerging evidence suggests that occult tumor-mediated autoimmunity may contribute to diagnostically challenging cases of IAD.^[5]^ IAD has been documented as a paraneoplastic syndrome driven by autoimmunity targeting corticotroph cells; yet, literature reviews indicate that such paraneoplastic presentations of IAD are rare. When they do occur, they are primarily associated with malignancies including small-cell lung cancer, thymic carcinoma, and neuroendocrine tumors. Notably, no clinical evidence has thus far established an association between IAD and testicular germ cell tumors, with no such cases reported in the current medical literature.

Paraneoplastic syndrome is defined as symptoms or signs arising from damage to organs/tissues distant from the primary malignant tumor and its metastatic sites, unrelated to direct tumor invasion, metastasis, or treatment-related side effects.^[6]^ Instead, its pathogenesis typically involves immune system abnormalities – such as cross-reactive antibodies attacking self-tissues – or the secretion of tumor-derived factors.^[7]^ Approximately 10% to 15% of cancer patients develop paraneoplastic syndromes, with most paraneoplastic neurological syndromes being immune-mediated.^[8]^ Paraneoplastic endocrine syndromes associated with non-endocrine tumors often occur in highly malignant neoplasms, but their onset lacks consistent correlation with tumor stage, malignant potential, or patient prognosis.^[9]^ Notably, immune attacks linked to paraneoplastic syndromes may slow tumor growth, resulting in slower progression and better prognosis compared to tumors unassociated with such syndromes. For instance, a case report described IAD concurrent with large-cell neuroendocrine carcinoma (LCNEC), where LCNEC was diagnosed 3 years after IAD onset; the patient maintained stable disease for over 4 years, with a relatively favorable prognosis.^[10]^

The pathogenesis of IAD as a paraneoplastic syndrome is associated with autoimmune cross-reactivity.^[11]^ Malignant tumors may induce the production of cross-reactive antibodies that target and destroy pituitary corticotrophs – a phenomenon most frequently observed in thymomas and small-cell lung cancer. Additionally, tumor-associated immune responses may trigger lymphocytic hypophysitis, leading to selective corticotroph damage. A case of IAD complicated by LCNEC further supported this mechanism: cancerous tissues showed ectopic proopiomelanocortin (POMC) expression and lymphocytic infiltration, and circulating autoantibodies against POMC were detected, confirming IAD as a paraneoplastic syndrome in this patient.^[12]^ POMC conversion to ACTH requires prohormone convertases and mature secretory vesicles. Due to poor differentiation, some non-pituitary tumors fail to fully establish this pathway, preventing proper processing of POMC into ACTH.^[13]^ For example, silent POMC expression has been observed in carcinoid tumors without ectopic ACTH syndrome. The Ki-67 labeling index, a well-validated biomarker of cell proliferation, correlates positively with tumor proliferative capacity and inversely with histological differentiation grade.^[14,15]^ In our patient with IAD and testicular germ cell tumor, immunohistochemistry (Fig. 2) showed a Ki-67 index of 20%, positive staining for SALL4 and PLAP (confirming poorly differentiated intratubular germ cell neoplasia in situ), and negative ACTH staining (ruling out ectopic ACTH expression). Given the tumor’s poor differentiation, we hypothesize it may express POMC but fail to process it into ACTH, thereby inducing anti-POMC antibodies that attack pituitary corticotrophs. Regrettably, we were unable to test for these autoantibodies to verify this mechanism.

Beyond paraneoplastic autoimmunity, the pathogenesis of IAD remains incompletely understood. Congenital IAD, which primarily affects pediatric populations, is linked to pathogenic variants in genes such as POMC (encoding the precursor of ACTH, which governs ACTH production via proteolytic processing) and TBX19 (a pituitary-specific transcription factor that regulates POMC expression, corticotroph maturation, and HPA axis function).^[16]^

Acquired IAD is associated with multiple factors, among which autoimmunity is the most well-recognized, supported by 4 key lines of evidence: Most patients with acquired IAD have comorbid autoimmune diseases, most commonly hypothyroidism and Hashimoto thyroiditis, and may also present with type 1 diabetes or Crohn disease.^[17]^ IAD can manifest as a paraneoplastic syndrome, where ectopic expression of pituitary antigens in tumors disrupts ACTH-related immune tolerance. Immune checkpoint inhibitors, a class of novel anticancer agents, activate antitumor immunity but may also induce autoimmune damage to endocrine glands (thyroid, pituitary, adrenal), contributing to IAD.^[18]^ While pituitary MRI in most IAD patients shows no structural abnormalities, some present with an empty sella turcica – an observation that has led researchers to propose a potential autoimmune link. ^[19]^

Other etiological factors for acquired IAD include pituitary lesions (e.g., ischemic injury, lymphocytic hypophysitis); lymphocyte infiltration in the pituitary gland has been detected postmortem in an IAD patient. Long-term opioid use can inhibit endogenous endorphin production and ACTH synthesis, leading to IAD.^[20]^ Long-term alcohol abuse is also a rare etiological factor, as chronic alcoholism suppresses the HPA axis.^[21]^

IAD presents with diverse, nonspecific clinical manifestations, including fatigue, malaise, somnolence, nausea, vomiting, anorexia, weight loss, hypoglycemia, hyponatremia, and eosinophilia – symptoms often misattributed to gastrointestinal or neuropsychiatric disorders.^[22]^ A study found that ~64.71% of IAD patients initially seek care in gastroenterology departments, 11.76% in neurology/psychiatry departments, and only 23.53% directly in endocrinology departments.^[23]^ Many patients present only when developing life-threatening complications such as adrenal crisis or hypoglycemic coma, underscoring the critical importance of early diagnosis for improving outcomes.^[24]^

Our patient exhibited unique clinical features: he initially presented with alopecia areata, which showed no improvement after 6 months of dermatological treatment (with newly grown hair turning white). He later developed decreased libido, fatigue, and malaise but lacked classic IAD manifestations such as anorexia, nausea, vomiting, or weight loss. Notably, alopecia areata improved following IAD treatment. Alopecia areata is an immune-mediated disorder targeting hair follicles, associated with genetic susceptibility, immune dysregulation, and environmental triggers (e.g., psychological stress, smoking, alcohol, sleep disturbances).^[25]^ Infections, toxins, and diet may also contribute by disrupting autoimmune homeostasis.^[26]^ While no previous reports have linked alopecia areata to IAD, their shared autoimmune pathogenesis suggests a potential association cannot be excluded.

Additionally, our patient lacked common laboratory abnormalities of IAD: previous studies reported hyponatremia in 49.6% of cases, eosinophilia in 30.1%, and hypoglycemia in 10.6%,^[27]^ whereas our patient had normal sodium levels, no eosinophilia, and stable blood glucose. This atypical presentation posed diagnostic challenges but also provides valuable insights for clinicians.

IAD management relies on individualized hydrocortisone replacement therapy, tailored to clinical manifestations, symptom severity, and ACTH deficiency degree. For our patient, surgical resection of the testicular germ cell tumor was also performed given its malignant nature. Following treatment, the patient’s clinical symptoms improved significantly, the tumor was completely resected, and both the patient and his family expressed satisfaction with the outcomes.

This case has several limitations, primarily due to institutional resource constraints and socioeconomic factors: we were unable to perform critical diagnostic tests (e.g., ACTH stimulation test, POMC autoantibody detection) or complete comprehensive immunohistochemical staining due to reagent shortages. Additionally, genetic testing was not conducted to explore whether an underlying genetic syndrome links IAD and testicular germ cell tumor – an important gap for future investigation.

This case highlights 2 key clinical insights: first, clinicians should remain vigilant for rare cutaneous initial manifestations of IAD, such as alopecia areata; second, multisystem neoplastic screening is essential in adult-onset IAD to evaluate potential paraneoplastic etiologies. Notably, germ cell tumors may represent a previously underrecognized etiological factor in adult IAD. By expanding the phenotypic spectrum of IAD and providing novel mechanistic hypotheses, this case refines diagnostic paradigms and offers clinical guidance for managing similar challenging cases.

## Author contributions

**Conceptualization:** Ming Yang.

**Data curation:** Ming Yang, Dongting Fu.

**Formal analysis:** Ming Yang, Shuangzhu Lin.

**Funding acquisition:** Ming Yang.

**Investigation:** Ming Yang, Yu Wang.

**Methodology:** Ming Yang.

**Project administration:** Ming Yang.

**Resources:** Ming Yang, Xiaoli Wang.

**Software:** Ming Yang, Siyu Lu, Man Li.

**Supervision:** Ming Yang.

**Validation:** Ming Yang.

**Visualization:** Xiaoli Wang.

**Writing – original draft:** Ming Yang.

**Writing – review & editing:** Xiaoli Wang.
